# Quantitative and Qualitative Platelet Derangements in Cardiac Surgery and Extracorporeal Life Support

**DOI:** 10.3390/jcm10040615

**Published:** 2021-02-06

**Authors:** Enrico Squiccimarro, Federica Jiritano, Giuseppe Filiberto Serraino, Hugo ten Cate, Domenico Paparella, Roberto Lorusso

**Affiliations:** 1 Department of Cardiac Surgery, Mater Dei Hospital, 70125 Bari, Italy; e.squiccimarro@gmail.com; 2 Department of Emergency and Organ Transplant (DETO), University of Bari, 70125 Bari, Italy; 3 Cardio-Thoracic Surgery Department, Heart & Vascular Centre, Maastricht University Medical Centre (MUMC), 6229HX Maastricht, The Netherlands; robertolorussobs@gmail.com; 4 Cardiac Surgery Unit, Department of Experimental and Clinical Medicine, University “Magna Graecia” of Catanzaro, 88100 Catanzaro, Italy; serraino@unicz.it; 5 Center for Thrombosis and Hemostasis (CTH), University Medical Center of the Johannes Gutenberg-University Mainz, D-55131 Mainz, Germany; h.tencate@maastrichtuniversity.nl; 6 Thrombosis Center Maastricht, Maastricht University Medical Center (MUMC), 6229HX Maastricht, The Netherlands; 7 Cardiovascular Research Institute Maastricht (CARIM), 6229HX Maastricht, The Netherlands; 8 Division of Cardiac Surgery, Santa Maria Hospital, GVM Care & Research, 70125 Bari, Italy; domenico.paparella@uniba.it; 9 Department of Medical and Surgical Sciences, University of Foggia, 71122 Foggia, Italy

**Keywords:** platelet, thrombocytopenia, cardiac surgery, inflammation, biological prosthesis, extracorporeal life support

## Abstract

Thrombocytopenia and impaired platelet function are known as intrinsic drawbacks of cardiac surgery and extracorporeal life supports (ECLS). A number of different factors influence platelet count and function including the inflammatory response to a cardiopulmonary bypass (CPB) or to ECLS, hemodilution, hypothermia, mechanical damage and preoperative treatment with platelet-inhibiting agents. Moreover, although underestimated, heparin-induced thrombocytopenia is still a hiccup in the perioperative management of cardiac surgical and, above all, ECLS patients. Moreover, recent investigations have highlighted how platelet disorders also affect patients undergoing biological prosthesis implantation. Though many hypotheses have been suggested, the mechanism underlying thrombocytopenia and platelet disorders is still to be cleared. This narrative review aims to offer clinicians a summary of their major causes in the cardiac surgery setting.

## 1. Introduction

Platelets are anucleated blood components with a pivotal role in hemostasis and also other functions in the biology and pathophysiology of complex diseases [1]. Beyond hemostasis and thrombosis, contemporary knowledge ascribes to platelets a key role also in inflammation and innate immunity [2,3,4,5]. Therefore, platelets may be considered as immune cells [6]. Quantitative and qualitative platelet derangements represent a shortcoming in cardiac surgery and extracorporeal life supports (ECLS). Thrombocytopenia, indeed, is an intrinsic drawback of cardiac surgery with a prevalence > 30% [7,8]. It is not a trivial event but rather a clinically relevant entity independently associated with increased postoperative morbidity and mortality [7,8]. A conclusive knowledge about the causes of the phenomenon is lacking whereas certainty of its clinical implications exists [9].

**Figure 1:**
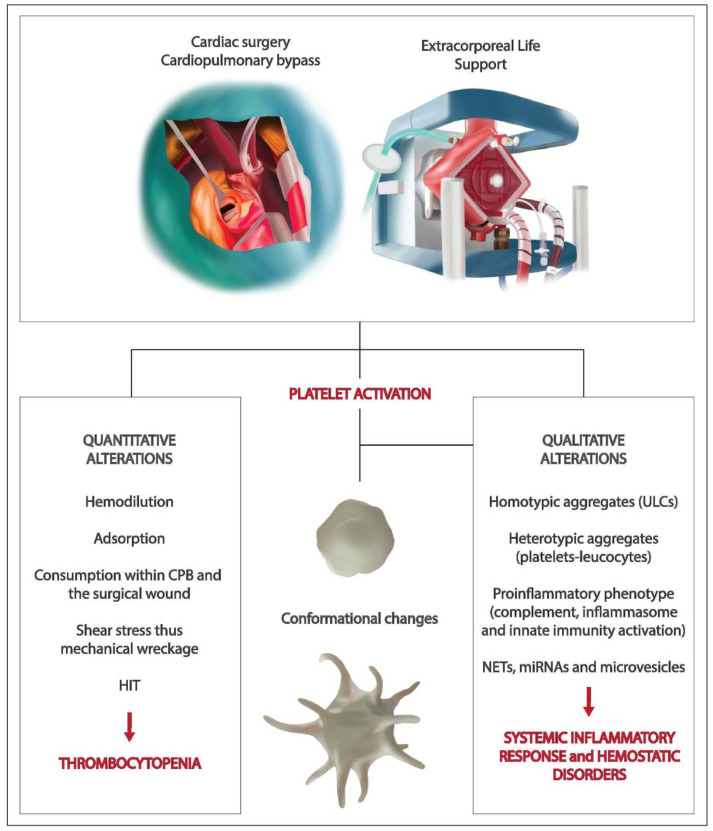
Platelet activation as a consequence of cardiopulmonary bypass-assisted cardiac surgery and extracorporeal life support. A summary of quantitative and qualitative platelet derangements. Abbreviations: cardiopulmonary bypass (CPB), heparin-induced thrombocytopenia (HIT), ultra-large antigenic complexes (ULCs), neutrophil extracellular traps (NETs), microRNAs (miRNAs).

Therefore, beyond the role of platelets in hemostasis and thrombosis, the present review aims to give a comprehensive analysis of platelet behavior in the cardiac surgery setting (Figure 1).

## 2. Platelets, Cardiac Surgery and Extracorporeal Circulation: The Axis of Hemostasis, Inflammation and Innate Immunity

Multiple sides of platelet biology greatly impact on heart procedures because cardiac surgery enhances a systemic immuno-inflammation response, platelet activation and the coagulation cascade [10].

Platelets undergo both quantitative and qualitative alterations throughout a cardiopulmonary bypass (CPB), the extracorporeal circulation applied to cardiac surgery procedures. The interaction between blood and artificial surfaces of the CPB triggers damage to several cells, the release of various inflammatory cytokines and the activation of the complement and coagulation-fibrinolysis systems [11,12]. During a CPB, platelet changes are caused by hypothermia, shear stress, extensive exposure to artificial surfaces and the use of exogenous drugs (heparin and protamine) [11,12]. Moreover, the coagulation cascade also begins with the activation of factor XII. Clotting factor activation occurs and initiates the subsequent activation of kallikrein, the kinin-bradykinin system and the fibrinolytic and complement cascades [11,12]. All of these mechanisms lead to increased postoperative outcomes such as mortality, major complications (e.g., stroke, acute kidney injury, postoperative infections) and a prolonged in-hospital length of stay [7]. Moreover, the hemodilution related to a CPB contributes to increasing the rate of thrombocytopenia [10,11,12]. Therefore, cardiac surgery and a CPB lead to a complex homeostatic alteration that enhances the so-called “thromboinflammation”, a complex mechanism involving inflammation, thrombosis and innate immunity [10,11]. The same scenario occurs also as a response to other triggers such as veno-arterial (V-A) and veno-venous (V-V) extracorporeal membrane oxygenation (ECMO) and cardiac prosthetic devices [1,13,14].

Moreover, a major role is attributed to the direct platelet-leucocyte interaction that bidirectionally boosts their reciprocal activation [15]. This cross-talk is fundamental in the multistep pathway of neutrophil extravasation (i.e., margination, rolling, extravasation and migration) that occurs in the systemic inflammatory response syndrome in patients on a CPB [16,17]. This process causes the activation of the endothelial cells and of several cellular adhesion molecules (CAMs) resulting lastly in tissue metabolic impairments and an ischemia-reperfusion injury (IRI) [16,17]. Moreover, the interplay between platelets and neutrophils was reported as a prerequisite for the release of neutrophil extracellular traps (NETs), which further triggers platelet activation and aggregation [18,19]. Furthermore, the binding of platelets’ integrin αIIbβ3 to neutrophils’ macrophage-1 antigen (Mac-1) stimulates the signaling leading to the formation of NETs. This interaction activates an inflammatory response mediated by the nuclear factor-kB (NF-kB) [20,21,22,23].

Platelets also modulate the immunoactivity of monocytes/macrophages by NF-kB activation. Moreover, the synthesis of proinflammatory mediators is stimulated. Platelets promote monocytes’ chemokines synthesis via P-selectin/P-selectin glycoprotein ligand (PSGL)-1 axis mediated “regulated on activation, normal T cell expressed and secreted” (RANTES) activation [24]. Furthermore, platelet α-granules (their most abundant storage granules) contain a diverse range of cytokines and chemokines among which are CXCL1, platelet factor 4 (PF4; CXCL4), CXCL5, interleukin-8 (IL-8) and RANTES [25]. Platelets have also been shown to independently enhance the inflammatory cascade in innate immune cells in vivo, thus contributing to the release of IL-1 cytokines [26].

In addition to these direct and indirect biological mechanisms, platelets also interact with the classical and the alternative pathways of the complement system [27]. The release of chondroitin sulfate modulates complement activity promoting anaphylatoxins and membrane attack-complex (MAC) generation, thus inducing further platelet activation [28,29]. The interplay between platelets and the complement system seems to involve platelet microparticles containing complement components such as C5b-9 at their surface [30,31].

Furthermore, platelets are the main source of microparticles in the bloodstream [32]. Extracellular vesicles composition varies and includes chemokines, cytokines and CAMs as well as small non-coding RNAs called microRNA (miRNA) [33,34]. MiRNA are involved in gene expression via negative post-transcriptional regulation [33,34]. Circulating miRNAs (i.e., miR-223 and miR-499) were detected following thrombin stimulation [34,35]. Even if the underlying mechanism is still to be cleared, miRNAs could transfer genetic material to recipient cells (among which are endothelial and immune cells) impacting the biological functions of recipient cells (i.e., regulating CAMs expression) [34,35]. Indeed, plasma exosomal miR-223 concentration was found to increase after CPB onset and to downregulate the inflammatory response reducing IL-6 and NLRP3 expression in monocytes [36]. A few studies have suggested that platelet microparticles may also be a source of a circulating tissue factor, explaining the activation of the extrinsic coagulation cascade and again linking hemostasis with immuno-inflammation via platelet activity [37].

Therefore, platelets contain abundant RNAs even if they lack a nucleus. Intraplatelet miRNA alterations may influence platelet messenger RNAs and consequently their proteome. Platelet protein expression impairment may further contribute to postoperative platelet dysfunction. A platelet qualitative impairment such as a reduced surface GPIb expression was found to be associated with the overexpression of some miRNAs (i.e., mir-10b and mir-96) and also with enhanced platelet Bax apoptotic signaling in cardiac surgery cohorts [38,39]. Microcirculatory impairment is another factor associated with platelet dysfunction following heart procedures. It consists of the loss of capillary density and increased flow heterogeneity and reflects how endothelial activation and glycocalyx degradation are both a consequence and a determinant of the systemic inflammatory response [40,41,42]. Furthermore, a recent investigation showed how perivascular mast cells were activated through the release of the lipid mediator platelet activating factor (PAF) from gut microvascular endothelial-adherent platelets to explain the inflammatory mediated tissue damage and organ injury following a CPB [43]. This mechanism might highlight platelets as a direct determinant of IRI related tissue damage.

## 3. Platelets and Extracorporeal Membrane Oxygenation

ECMO is a temporary mechanical support for severe cardiac or respiratory failure or both [44,45]. Although the technology is almost identical, ECMO and a CPB differ in many aspects such as the setting of the application, the duration of the support and the rates of complications. Despite fifty years of continuous improvement in technology and management, ECMO has weaknesses to be overcome [46]. Thrombocytopenia and platelet dysfunction are ECMO shortcomings whose underlying mechanisms remain not fully understood.

First, the alteration in the phenotype and thrombocytopenia of platelets could be the result of a huge inflammatory response. As in CPB patients, activated platelets circulate in the bloodstream of patients on ECMO following contact with the circuit’s artificial surfaces [47]. Blood exposure to non-physiological conditions promotes thrombotic events [47].

Secondly, the high-speed rotation of the centrifugal pump triggers a mechanical shear stress causing platelet dysfunction, rupture and the shedding of receptors [48]. In a recent ex vivo study, Sun and colleagues demonstrated the pivotal role of the oxygenator, pump and circuit in affecting platelet function [49]. Platelet receptor shedding and a persistent release in microparticles confirmed a deficient adhesion and platelet count reduction increasing over time [49]. Similarly, a decreased binding capacity of platelets was demonstrated by the loss of surface receptors [50,51,52]. Cannulation sites and components of the circuits have also been investigated to identify how they could alter platelet shape, function and count [53]. Fuchs and colleagues indicated the pump as the largest site risk for platelet activation followed by the reinfusion cannula and lastly the connectors [53].

Thirdly, as systemic anticoagulation is required, patients are likely to develop heparin-induced thrombocytopenia (HIT) [54]. The Extracorporeal Life Support Organization (ELSO) anticoagulation guideline recommends the administration of antithrombotic therapy during ECMO; unfractionated heparin (UFH) is widely administered [54,55]. The prevalence of HIT in adult ECMO patients is estimated at 3.7% with a similar prevalence between V-A and V-V ECMO [55,56]. However, this result could be biased by prompt HIT recognition and switching to alternative anticoagulants [56]. Frequently, physicians treat a patient by just thinking about HIT and without a solid diagnosis. Therefore, an agreed international protocol is strongly required for the early identification and univocal management of HIT patients in ECMO.

Other speculations have been advanced to justify thrombocytopenia during the ECMO run [57,58,59].

A few studies have focused attention on the duration of support and platelet count decrease [57,58,59]. Ang and colleagues showed how time on ECMO directly induced thrombocytopenia and the need for blood product transfusions, particularly platelets [60]. However, several factors such as a pre-existent disease (i.e., sepsis and postcardiotomy cardiogenic shock), antiplatelet drugs and bleeding events could have biased the authors’ results. Panigada and co-workers observed similar outcomes in their cohort study [58]. Abrams first stated that platelet count reduction was mostly due to the severity of the disease of patients rather than related to the duration of ECMO support [57]. In a recent meta-regression, we confirmed that a worsening in thrombocytopenia was independent of the number of days on ECMO [56]. However, we speculated that after few days the inflammatory response induced by ECMO might not affect the freshly generated platelets due to the beginning of an endothelization mechanism of the circuit [56,61,62].

The ECMO mode also does not seem to influence the degree of thrombocytopenia [56]. After analyzing the available evidence in the literature, we estimated a comparable platelet count decrease in almost 25% and 23% of adult V-A and V-V ECMO subjects, respectively [56].

However, in addition to the causes, future research should focus on the possible clinical implications of platelet disorders (i.e., hemorrhagic or thrombotic events, blood product transfusions and mortality). Currently, the literature lacks trials that centrally address this issue. Researchers will have to face the multiple biases (i.e., pre-existing diseases, anticoagulation and antiplatelet drugs, sepsis) that make the investigation a real challenge.

## 4. Platelets and Aortic Biological Prosthesis

In the past years, physicians have had to cope with periprocedural thrombocytopenia in patients receiving an aortic biological prosthesis [63]. The discussion has focused on blood interaction with the artificial valves and the possible consequences [63]. An increased platelet turnover and destruction plays an important role [63]. Moreover, a major bleeding risk after an aortic valve replacement is not an uncommon event ranging from 4% for surgical tissue valves to 16% for the transcatheter prosthesis [63]. The shear stress through an artificial device has been suggested as a central mechanism of hemostatic dysfunction [64,65]. It can induce conflicting mechanisms at the same time; platelet activation, aggregation and generation of procoagulant microparticles as well as platelet dysfunction, loss of surface receptors and bleeding complications [64,65]. Furthermore, patients receiving surgical bioprostheses are exposed to the adverse effect of the CPB [54]. The CPB duration seems to be a major determinant for the development of postoperative thrombocytopenia [66]. However, this mechanism could change according to the different type of valve prosthesis implanted.

In 2006, Le Guyader and colleagues observed platelet activation after aortic valve replacements with two kinds of mechanical valves and three kinds of tissue valves [67]. They found platelet activation in all of the prostheses on the eighth postoperative day (POD) and was still present in the bioprosthesis group at the two month follow-up [67]. Ravenni and co-workers reported a decrease in platelet counts in different bioprosthesis types on the first POD; a stentless bioprosthesis showed a significant decrease in the postoperative platelet count compared with stented tissue valves [68]. Similarly, in 2016 Stanger and colleagues reported a significant decrease in the postoperative platelet count in three types of bioprostheses [69]. Nevertheless, those tissue valves were not associated with bleeding complications [69].

Several investigators have reported stentless bioprostheses as risk factors for postoperative thrombocytopenia [70,71,72,73]. Although showing a good hemodynamic performance, stentless bioprosthesis implantation has been associated with postoperative thrombocytopenia [63]. However, a decreased postoperative platelet count did not affect the postoperative outcomes compared with stented bioprostheses except for an increased rate of red blood cell transfusions [63]. The postoperative platelet count decreased from 60% to 77% after a stentless bioprosthesis implantation [63]. Likewise, the platelet count fell from 53% to 60% after the implantation of rapid deployment valves (RDVs) [63]. Like the stentless valves, thrombocytopenia after RDV implantation seemed to occur in the first PODs before a slow recovery within 7 to 10 days after surgery [69,74,75,76]. The origin of thrombocytopenia after RDV implantation is still unknown. To explain the phenomenon in RDVs, investigators have advanced analogous hypotheses to those for thrombocytopenia after the implantation of stentless bioprostheses [74,75,76,77].

Interestingly, thrombocytopenia and platelet disorders do not only occur after the implantation of surgical bioprostheses [63]. Several papers have reported that transcatheter aortic valve implantation (TAVI) patients have also experienced a temporary platelet count decrease following the procedure [63,78,79,80,81,82,83,84,85,86,87,88,89,90]. The platelet count drop ranged from 21% to 72% after TAVI with associated adverse outcomes [63,89,91]. The cause was most likely multifactorial; the valve design, the shear stress, the valve size, the length and type of the procedure and the amount of low-osmolar contrast agents used can interact together and elicit platelet destruction and increase the coagulation cascade and the inflammatory process leading to thrombocytopenia [63,89,90]. Moreover, mispositioning and TAVI migration are predictors of a platelet count decrease supporting the hypothesis of shear stress in the origin of thrombocytopenia [12]. Furthermore, thrombocytopenia occurs more frequently in patients with balloon-expandable valves (BEVs) [63,85,87,90]. Considering the prosthesis shape, the use of large sheaths, pre-dilatation and surgical cut-down for femoral access, a BEV was identified as a new predictor of TAVI related thrombocytopenia [63,85,87,90]. Furthermore, TAVI related thrombocytopenia has been found to be associated with increased early and overall mortality after TAVI [63,89].

Future investigations should focus on defining thrombocytopenia and platelet disorders after the implantation of biological prosthesis to improve patient management and to reduce adverse events.

## 5. Heparin-Induced Thrombocytopenia

Heparin-induced thrombocytopenia (HIT) is a rare drawback after the exposure to either unfractionated heparin (UFH) or low molecular weight heparin (LMWH). Heparin is a worldwide used anticoagulant because of its efficiency, accessibility, reversibility and costs beyond compare.

HIT is characterized by a decreased platelet count of about 30–50% leading to severe thrombocytopenia (<100 × 10^9^/L). However, typical factors such as hemodilution must be ruled out in the diagnostic phase. Hemorrhagic complications are rare whereas thromboembolic events occur more frequently (up to ~ 50% if not diagnosed/treated) and might jeopardize the patient’s outcome [92]. HIT is estimated to occur once in 1500 hospitalizations in the United States with high-risk cardiac surgery patients [92]. Furthermore, HIT is associated with a four-fold higher in-hospital mortality, three-fold longer median hospitalization time and four-fold higher costs of hospitalization compared with thrombocytopenia from other etiologies [93].

HIT’s risk factors are both host and drug related; the female gender has been linked to a doubled risk of developing HIT [94] while a younger age (<40 years) seems to entail a milder risk [95]. Furthermore, recent genome-wide association studies (GWAS) have highlighted candidate gene variants (i.e., the HLA-DRB3*01:01 allele) associated with a risk of HIT [96,97,98]. Among the drug related factors, the duration of heparin exposure plays a major role with a shorter span carrying a lower risk [99]. UFH therapy is more likely to lead to HIT compared with LMWH [100,101,102,103]. Platelet factor 4 (PF4), indeed, forms oligomers binding multiple UFH oligosaccharides that further share several PF4 tetramers, leading to the assembly of ultra-large antigenic complexes (ULCs) [60,104,105]. Bovine heparin may carry a higher risk for HIT compared with porcine heparin [106,107].

HIT is a serious immune mediated adverse reaction to heparin polyanions caused by pathogenic immunoglobulin G (IgG) antibodies. IgGs bind to complexes of heparin and PF4, a cationic chemokine stored within platelet alpha granules characterized by a high affinity for anionic molecules. These complexes are optimally formed at a stoichiometric concentration (molar ratio 1:1). Noxae such as major surgery, a CPB and mechanical circulatory supports (MCSs) (e.g., ECMO and ventricular assist devices (VADs)) can trigger massive platelet activation and subsequent additional PF4 release [108]. ULCs consequently cross-link the surface receptors of platelets (FCγIIa) leading to a switch toward a hyper-aggregative phenotype [19,109,110]. The process promotes a complex cascade of events involving endothelial cells, immune cells and NETs and coagulation factors and other circulating molecules [19,109,110].

The mutual effect of pre-existent vascular stress, incessant platelet activation and the need of profound heparinization make adult cardiac surgery the most prone setting for HIT occurrence (50% to 70%) [111,112,113,114]. Conversely, pediatric patients show lower rates of seroconversions (0–2%) most likely due to the absence of chronic vascular insults [91]. Despite the recurrence of anti-PF4/heparin antibodies in heart surgery patients, only 2–3% develop HIT [115]. The detection of antibodies is required to diagnose HIT. In addition to the low platelet count, the “4 Ts” score is a bedside tool used to estimate the probability of HIT that improves the specificity of antibody tests in cardiac patients (Table 1) [116].

Moreover, preoperative evidence of anti-PF4/heparin antibodies is reported in 5% to 22% of cardiac patients [117]. However, an uncertain prognostic value is attributed to this finding because of the controversial data about the preoperative positivity to anti-PF4/heparin antibodies and postoperative HIT, thrombosis and an adverse outcome [19,118,119,120,121]. Therefore, routine screening for such antibodies is discouraged (unless clinically evident HIT signs are manifested or in cases of a history of HIT).

HIT occurrence makes heart surgery a challenging event that can counteract the results of even the most successful operation. This eventuality should imply the deferral of any elective procedure until the results of laboratory tests are available. However, cardiac surgery is a practice in which deferring a procedure is not always a feasible option. Hence, several alternative strategies exist; pharmacological agents such as direct thrombin inhibitors (DTIs), factor Xa inhibitors (i.e., fondaparinux) and platelet inhibitors (i.e., prostaglandins) are plausible options.

Among DTIs, bivalirudin is the most used alternative agent in cardiac surgery showing a comparable efficiency and similar perioperative morbidity rates with heparin [122,123]. Furthermore, while DTIs are usually monitored by activated partial thromboplastin time (aPTT), the activated clotting time (ACT) appeared to be reliable in monitoring anticoagulation with bivalirudin during a CPB [121]. Other DTIs such as argatroban and danaparoid are not recommended as alternative therapies during a CPB [124,125]. Several studies have reported bleeding after therapy discontinuation and unexpected thrombosis (ACT > 500 s) [124,125]. While HIT treatment with either fondaparinux or other molecules (i.e., epoprostenol, iloprost) is almost anecdotal [126], off-pump procedures and a plasma exchange represent other controversial approaches to minimizing heparin exposure [127,128,129,130].

## 6. Conclusions

Platelet functional or structural alterations are not epiphenomena of the acute reaction to CPB-assisted cardiac surgery, biological bioprosthesis and ECMO but rather have a major role in the thread linking hemostasis, inflammation and innate immunity. The multiple direct/indirect mechanisms that regulate this connection are still partially unclear. However, further studies addressing the pathobiological dynamics concurring to the abovementioned quantitative (i.e., thrombocytopenia) and qualitative (i.e., platelet activation and a shift towards a proinflammatory phenotype) platelet derangements in a cardiac surgery setting and ECLS may provide new insights possibly leading to improved patient outcomes.

## Figures and Tables

**Table 1:** Estimating the pre-test probability of HIT: the “4 Ts”.

“4 Ts” Category	2 Points	1 Point	0 Points
Thrombocytopenia	Platelet count fall > 50% AND platelet nadir ≥ 20 × 10^9^/L	Platelet count fall 30–50% OR platelet nadir 10–19 × 10^9^/L	Platelet count fall < 30% OR platelet nadir < 10 × 10^9^/L
Timing of platelet count fall	Clear onset between days 5–10 OR platelet fall ≤ 1 day (prior heparin exposure within 30 days)	Consistent with days 5–10 fall but not clear; onset after day 10 OR fall ≤ 1 day (prior heparin exposure 30–100 days ago)	Platelet count fall < 4 days without recent exposure
Thrombosis or other sequelae	New thrombosis OR skin necrosis; acute systemic reaction postintravenous heparin bolus	Progressive or recurrent thrombosis or non-necrotizing (erythematous) skin lesions or suspected thrombosis (not proven)	None
Other causes for thrombocytopenia	None apparent	Possible	Definite

## Data Availability

Not applicable.
